# Direct Exposure to Ethanol Disrupts Junctional Cell-Cell Contact and Hippo-YAP Signaling in HL-1 Murine Atrial Cardiomyocytes

**DOI:** 10.1371/journal.pone.0136952

**Published:** 2015-08-28

**Authors:** Kanako Noritake, Toshihiko Aki, Takeshi Funakoshi, Kana Unuma, Koichi Uemura

**Affiliations:** Department of Forensic Medicine, Graduate School of Medical and Dental Sciences, Tokyo Medical and Dental University, Tokyo, Japan; Albert Einstein College of Medicine, UNITED STATES

## Abstract

Direct exposure of cardiomyocytes to ethanol causes cardiac damage such as cardiac arrythmias and apoptotic cell death. Cardiomyocytes are connected to each other through intercalated disks (ID), which are composed of a gap junction (GJ), adherens junction, and desmosome. Changes in the content as well as the subcellular localization of connexin43 (Cx43), the main component of the cardiac GJ, are reportedly involved in cardiac arrythmias and subsequent damage. Recently, the hippo-YAP signaling pathway, which links cellular physical status to cell proliferation, differentiation, and apoptosis, has been implicated in cardiac homeostasis under physiological as well as pathological conditions. This study was conducted to explore the possible involvement of junctional intercellular communication, mechanotransduction through cytoskeletal organization, and the hippo-YAP pathway in cardiac damage caused by direct exposure to ethanol. HL-1 murine atrial cardiac cells were used since these cells retain cardiac phenotypes through ID formation and subsequent synchronous contraction. Cells were exposed to 0.5–2% ethanol; significant apoptotic cell death was observed after exposure to 2% ethanol for 48 hours. A decrease in Cx43 levels was already observed after 3 hours exposure to 2% ethanol, suggesting a rapid degradation of this protein. Upon exposure to ethanol, Cx43 translocated into lysosomes. Cellular cytoskeletal organization was also dysregulated by ethanol, as demonstrated by the disruption of myofibrils and intermediate filaments. Coinciding with the loss of cell-cell adherence, decreased phosphorylation of YAP, a hippo pathway effector, was also observed in ethanol-treated cells. Taken together, the results provide evidence that cells exposed directly to ethanol show 1) impaired cell-cell adherence/communication, 2) decreased cellular mechanotransduction by the cytoskeleton, and 3) a suppressed hippo-YAP pathway. Suppression of hippo-YAP pathway signaling should be effective in maintaining cellular homeostasis in cardiomyocytes exposed to ethanol.

## Introduction

Alcoholic cardiomyopathy, cardiac damage due to the chronic excessive drinking of alcoholic beverages, typically presents as idiopathic dilated cardiomyopathy including enlargement of the ventricle and resultant impairment of heart circulation [1, 2]. Acute drinking of excessive alcoholic beverages also causes severe cardiac damage, which includes cardiac arrhythmia, tissue injuries such as apoptosis, and ultimate heart failure [1]. Although the loss of cardiac function by acute alcohol intoxication results from the depression of nerve and pulmonary systems, direct ethanol toxicity on cardiomyocytes has also been demonstrated *in vitro* to include contractile dysfunction, hypertrophic cell growth, and the apoptotic death of cardiomyocytes [3, 4]. Lethal blood ethanol concentration on human beings may be around 0.4%, but cardiomyocyte apoptosis has been observed when the cells were exposed to more than approx. 1% ethanol [3].

Cell-cell communication is important for cellular homeostasis, especially for cardiomyocytes [5, 6]. Cardiomyocytes are connected end-to-end to each other through intercalated disks (ID), which consist of gap junctions (GJs), adherens junctions, and desomosomes [5]. Cardiomyocytes communicate electrically with each other by exchanging ions and small molecules through the GJ, as well as mechanically by transmitting actomyosin tension through the desmosome [5]. Connexin43 (Cx43), N-cadherin, and desmoglein2 are the main components of the GJ, adherens junction and desmosome, respectively. Cx43 is a quick turnover protein with a half-life of several hours *in vivo* [7]. Therefore, Cx43 turnover is tightly regulated through protein degradation systems such as ubiqutin-proteasome, endosome-lysosome, and autophagy [8].

Fully differentiated cardiomyocytes proliferate poorly after the establishment of sarcomeric actomyosin and intercellular connection through the ID [5]. The hippo pathway has been suggested as a regulator of proliferation and size-control of cardiomyocytes [9]. The hippo pathway is an evolutionarily conserved signaling pathway by which cell-cell contact is transferred to the nucleus, thereby regulating proliferation to maintain proper cell density; the hippo pathway is activated after an organ or tissue is fully developed [10, 11]. In mammals, core machinery of the hippo pathway comprises serine/threonine kinases Mst1/2, LATS1/2, and transcriptional coactivators YAP and TAZ, corresponding to *Drosophila* kinases Hippo, Warts, and *Drosophila* transcriptional co-activator Yorkie, respectively [10]. In addition to its physiological role, a recent report has suggested that hippo-YAP signaling is involved in the pathogenesis of cardiac arrhythmogenicity; a loss-of-function mutation in desmoglein2 leads to the loss of other components of the ID, which results in the activation of hippo-YAP signaling and subsequent adipogenesis [12].

Although the importance of the hippo pathway to the heart has been suggested under physiological as well as pathophysiological conditions, its involvement in alcoholic cardiac damage has not been examined to date. We thus examined the status of cell-cell communication, cellular cytoskeletal organization, and the hippo-YAP pathway in HL-1 cardiomyocytes exposed directly to ethanol.

## Material and Methods

### Materials and cell culture

HL-1 mouse atrial cardiomyocyte-derived cells, kindly provided by Dr. William C. Claycomb (Louisiana State University Medical Center) [13], were cultured on zelatin/fibronectin-coated dishes at 37°C in a humidified atmosphere containing 5% CO_2_ in Claycomb medium supplemented with 10% fetal bovine serum, 100 U/ml penicillin, 100 μg/ml streptomycin, 0.1 mM norepinephrine, and 2 mM L-glutamine. Once the cells had grown to confluence and started spontaneous beating, the indicated concentrations of ethanol were added directly to the medium. The culture dishes were sealed with laboratory film throughout the incubation period to minimize the evaporation of ethanol. Antibodies used in this study were anti-cleaved caspase-3 (#9661, Cell Signaling, USA), anti-caspase-9 (#9504, Cell Signaling), anti-p-JNK (Thr183/Tyr185-phosphorylated JNK, #4668, Cell Signaling), anti-total-JNK (#9528, Cell Signaling), anti-Cx43 (#MAB3067, Millipore, USA or #71–0700, Invitrogen, USA), anti-Cx40 (#sc-20466, Santa Cruz, USA), anti-Cx45 (#40–7000, Invitrogen), anti-LC3 (#2775, Cell Signaling), anti-desmoglein1/2 (#61002, Progen, Germany), anti-N-cadherin (#610920, BD Biosciences, USA), anti-p-Mst1/2 (Thr183-phosphorylated Mst1/Thr180-phosphorylated Mst2, #3681, Cell Signaling), anti-p-LATS1 (Ser909-phosphorylated LATS1, #9157, Cell Signaling), anti-p-YAP (Ser127-phosphorylated YAP, #4911, Cell Signaling), anti-total-YAP (#14074, Cell Signaling), anti-actin (#A2006, Sigma, USA), anti-GAPDH (#MAB374, Millipore) and anti-hsc70 (#sc-7298, Santa Cruz).

### Evaluation of cytotoxicity and viability

Cells were incubated with ethanol for 48 hours, and stained with 1 μM hoechst33342 and 1 μM PI for 10 min to visualize nuclear morphology and plasma membrane integrity, respectively. The percentage of apoptotic cells was calculated as the ratio of condensed nuclei to total nuclei. The percentage of PI-positive cells was calculated as the ratio of PI-positive cells to hoechst-positive cells. Cell viability was assessed by a modified MTT assay using a Cell Counting Kit-8 (CCK-8; Dojindo, Kumamoto, Japan).

### Western blot analysis

Western blot analysis was performed as described previously [14]. In brief, cells grown on 3 cm diameter dishes were detached from the dish with a scraper, suspended in STE buffer (0.32 M sucrose, 10 mM Tris-HCl, pH 7.4, 5 mM EDTA, 50 mM NaF, 2 mM Na_3_VO_4_, and protease inhibitor cocktail), disrupted by a sonicator (Sonifier 150, Branson, USA), and measured for protein concentration by the method of Bradford [15]. Equal amounts of protein were subjected to SDS-PAGE, transferred to a PVDF membrane, and blocked in TBS-Tween (150 mM NaCl, 10 mM Tris-HCl, pH 7.4, 0.05% Tween 20) containing 3% skim milk. The membrane was then incubated with appropriate antibodies, and then further with peroxidase-conjugated secondary antibodies. Antigens were visualized using a Western Lightning Chemiluminescence Reagent Plus Kit (Perkin Elmer Life Science, USA), and the band intensities were quantitated with an image analyzer (CS analyzer; Atto, Tokyo, Japan).

### Quantitative real-time PCR

Total RNA was isolated from the cells using TRIzol reagent (Invtrogen, USA), and cDNA was synthesized using oligo(dT)_15_ and SuperScriptII reverse transcriptase (Invitrogen). Quantitative real-time PCR (qPCR) was performed with the StepOnePlus real-time PCR system (Applied Biosystems, USA). The following primers were used for the amplification of templates: 5’-TTTGGCGTGCCGGCTTCACT-3’ and 5’-CTTCCCTCCGGCCGTGGAGT-3’ for Cx43; 5’-GTGCAGTGCCAGCCTCGTCC-3’ and 5’-GCCACTGCAAATGGCAGCCC-3’ for GAPDH. PCR was performed at 95°C for 3 sec. and 60°C for 30 sec. for 40 cycles. GoTaq Green Master Mix for quantitative PCR (Promega, USA) was used for amplification.

### Dye transfer assay

Scrape-loading dye transfer was performed to evaluate gap junctional cell-cell coupling [16]. HL-1 cells were treated with or without 2% ethanol for 48 hours. After the cells were washed with PBS, a scrape was made with a sterile pipette tip and the cells were incubated in the presence of 1 mg/ml Lucifer Yellow (Sigma Aldrich) in PBS for 5 minutes. Then the cells were washed with PBS and fixed with 4% paraformaldehyde in PBS. Cells containing Lucifer Yellow were observed under a fluorescence microscope (BZ-8100, Keyence, Osaka, Japan) and the fluorescent area was quantified using ImageJ software (National Institutes of Health).

### Transfection of vectors and fluorescence microscopy

Plasmid vectors expressing Cx43-mApple or mGFP-Cx43 (provided by Dr. Mattias M. Falk, Lehigh University) [17], LAMP1-mGFP (provided by Dr. Esteban C. Dell'Angelica, University of California Los Angeles) [18], LAMP1-RFP (Provided by Dr. Walther Mothes, Yale University) [19] (Addgene plasmid 1817), LMP2-GFP (provided by Dr. Jacques Neefjes, The Netherlands Cancer Institute) [20], GFP-Cx43Y286A (provide by Dr. Henrique Girao, University of Coimbra, Portugal) [21], and vimentin-PSmOrange (provided by Dr. Vladislav Verkhusha, Albert Einstein College of Medicine) [22] (Addgene plasmid 31922) were mixed with Lipofectamine2000 (Invitrogen) and added to the medium, and the samples were incubated overnight. After exposure to 2% ethanol for the indicated time periods, the cells were observed under a fluorescence microscope (BZ-8100, Keyence, Osaka, Japan).

### Cytochemistry and immunocytochemistry

HL-1 cells grown on cover slips were fixed with 4% paraformaldehyde for 15 min. After being washed with PBS, the cells were permeabilized by incubation with 0.5% Triton X-100/PBS for 5 min, and washed with PBS. For myofibril staining, the cells were incubated with 10 μg/ml Phalloidin-Tetramethylrhodamine B isothiocyanate (#P1951, Sigma Aldrich) in PBS for 10 min at RT. For YAP staining, the cells were incubated with anti-total-YAP antibody overnight at 4°C, then with Alexa488-conjugated anti-IgG antibody (Molecular probes, USA), and observed under a confocal fluorescence microscope (Nikon Eclipse Ti, Nikon, Tokyo, Japan) or a conventional fluorescence microscope (BZ-8100, Keyence).

### Statistical analysis

The Tukey-Kramer statistical method was used for multiple comparisons of more than three experimental groups. Student’s t-test was used for comparisons of two groups. Data are expressed as the mean ± S.E. of at least three samples. P values <0.05 were considered statistically significant.

## Results

### Ethanol dose-dependently induces apoptosis in HL-1 cells

**Fig 1:**
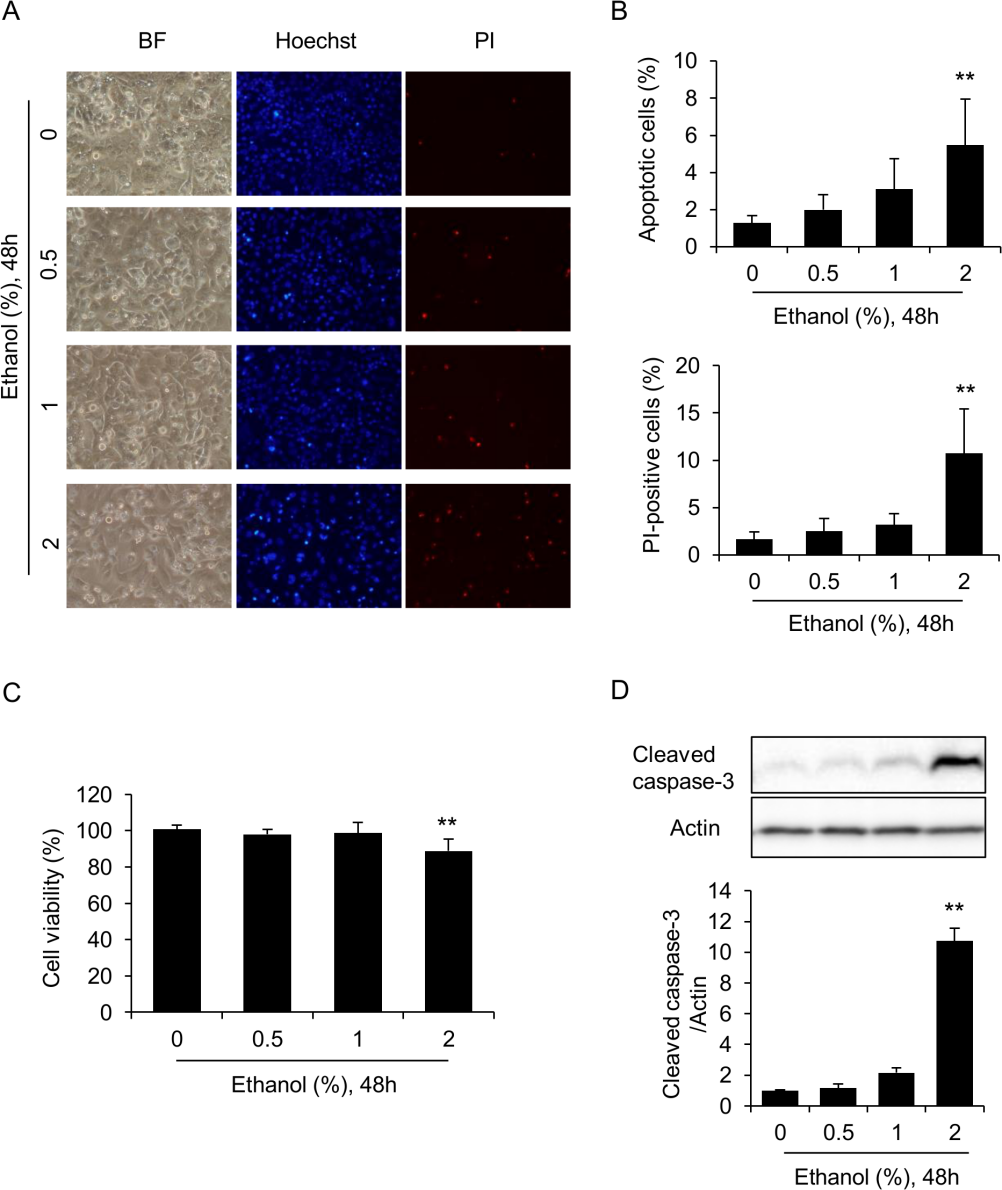
HL-1 cell death is induced after 48 hours exposure to 2% ethanol. (A and B) Direct exposure to ethanol induces nuclear chromatin condensation and plasma membrane injury in HL-1 cells. The cells were incubated in medium containing 0, 0.5, 1, or 2% ethanol for 48 hours, stained with Hoechst and PI fluorescence dyes, and observed under light as well as fluorescence microscopy (A). BF, bright field image. Percentages of cells showing nuclear chromatin condensation (apoptotic cells) and cells positive to PI staining (PI-positive cells) (B). (C) Ethanol decreases cell viability. Cellular viabilities were determined by MTT assay. Mean cell viability of untreated cells (0% ethanol) was set to 100%, and relative cell viabilities of ethanol-treated cells are shown. (D) Ethanol activates caspase-3. The cleaved form of caspase-3 (15 kDa, activated caspase-3) was identified by western blot analysis. Actin was also identified as a loading control. Relative levels of cleaved caspase-3 to actin were densitometrically determined. Each graph shows mean±S.E. (n = 3). **, p<0.01 versus 0%.

We first examined whether apoptosis is induced by exposing HL-1 cells to ethanol. Cells were treated with 0, 0.5, 1, or 2% of ethanol for 48 hours, and then assessed for nuclear morphology and plasma membrane damage. After exposure to 2% ethanol for 48 hours, the percentage of cells showing condensed nuclei (apoptotic cells) was significantly increased compared to control cells (Fig 1A and 1B), and approximately 10% of cells were PI-positive (dead cells) (Fig 1A and 1B). In correlation with the increased percentage of dead cells, cell viabilities decreased by about 10% after exposure to 2% ethanol for 48 hours (Fig 1C). To examine the activation of caspases, we evaluated the levels of the cleaved (activated) form of caspase-3. Western blot analysis indicated that the proteolytic activation of caspase-3 was increased by about 2-fold in cells treated with 1% ethanol, and by about 10-fold in cells treated with 2% ethanol, compared to 0% ethanol (Fig 1D). Thus, the direct addition of ethanol to the medium results in the apoptotic death of HL-1 cells after 48 hours of exposure.

### Ethanol activates caspases in a time-dependent manner

**Fig 2:**
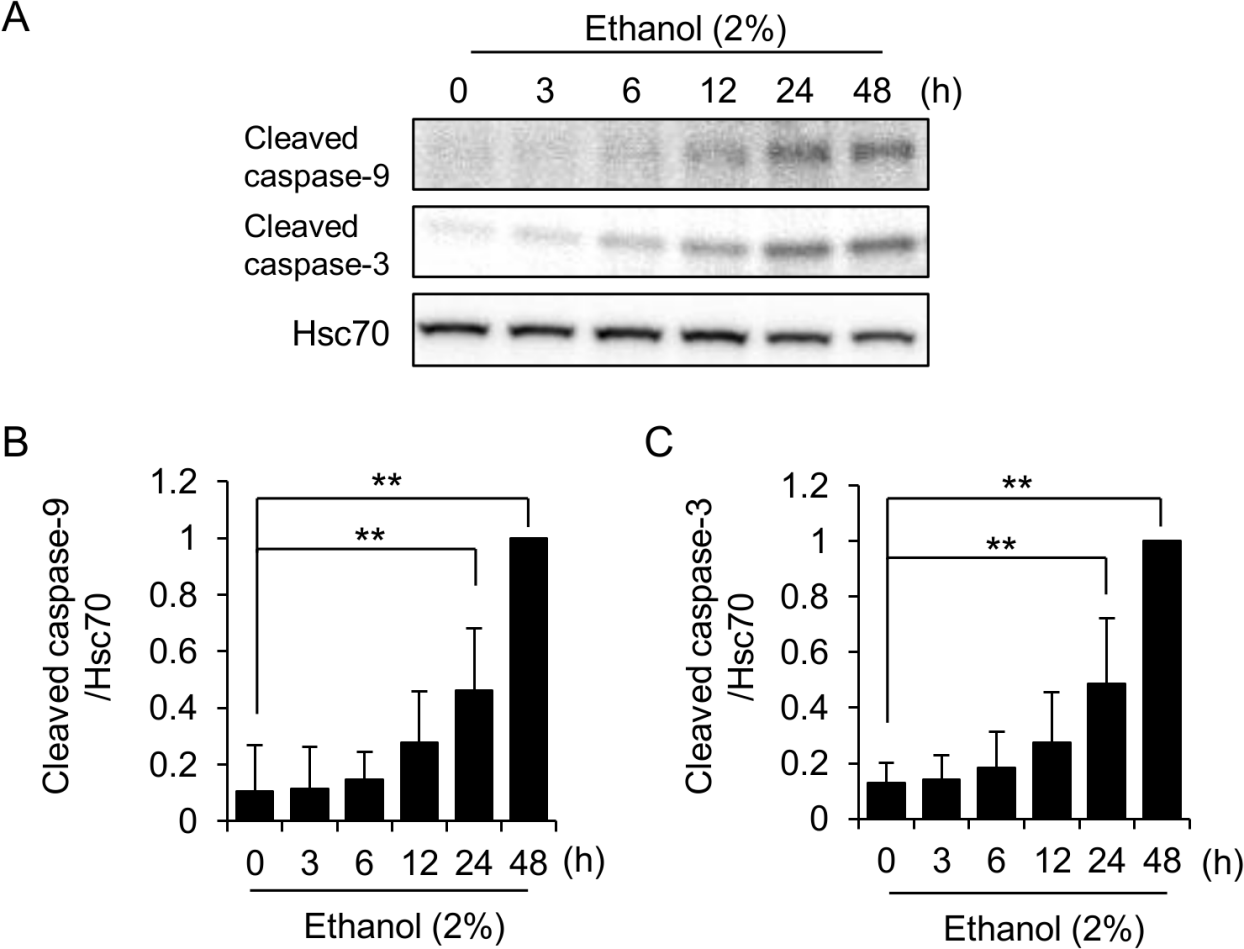
Time-dependent activation of caspases in ethanol-exposed HL-1 cells. (A) Activation of caspases in HL-1 cells occurs after 24–48 hours of exposure to 2% ethanol. Cells were incubated in medium containing 2% ethanol for the indicated time periods, and the levels of the cleaved (activated) forms of caspase-9 and -3 were determined by western blot analysis. Hsc70 served as a loading control. Quantitative analysis of cleaved caspase-9 (B) and -3 (C) is shown. Relative levels to ethanol (48 h)-treated group were shown. Each graph shows mean±S.E. of 6 samples. **, p<0.01.

We next examined the time course of caspase activation during ethanol treatment. Since we observed an incubation time-dependent decrease and increase of actin and GAPDH levels, respectively, under our experimental conditions (data not shown), we adopted hsc70 as an internal standard for time-course analysis. Activation of caspase-9 by exposure to 2% ethanol was observed after 24 hours, and the activation increased further for ~48 hours (Fig 2A and 2B). Essentially, the same time-dependency was also observed for the activation of caspase-3 (Fig 2A and 2B), suggesting the progression of an intrinsic apoptotic program.

### Both the mRNA and protein levels of Cx43 are down-regulated during exposure of HL-1 cells to ethanol

**Fig 3:**
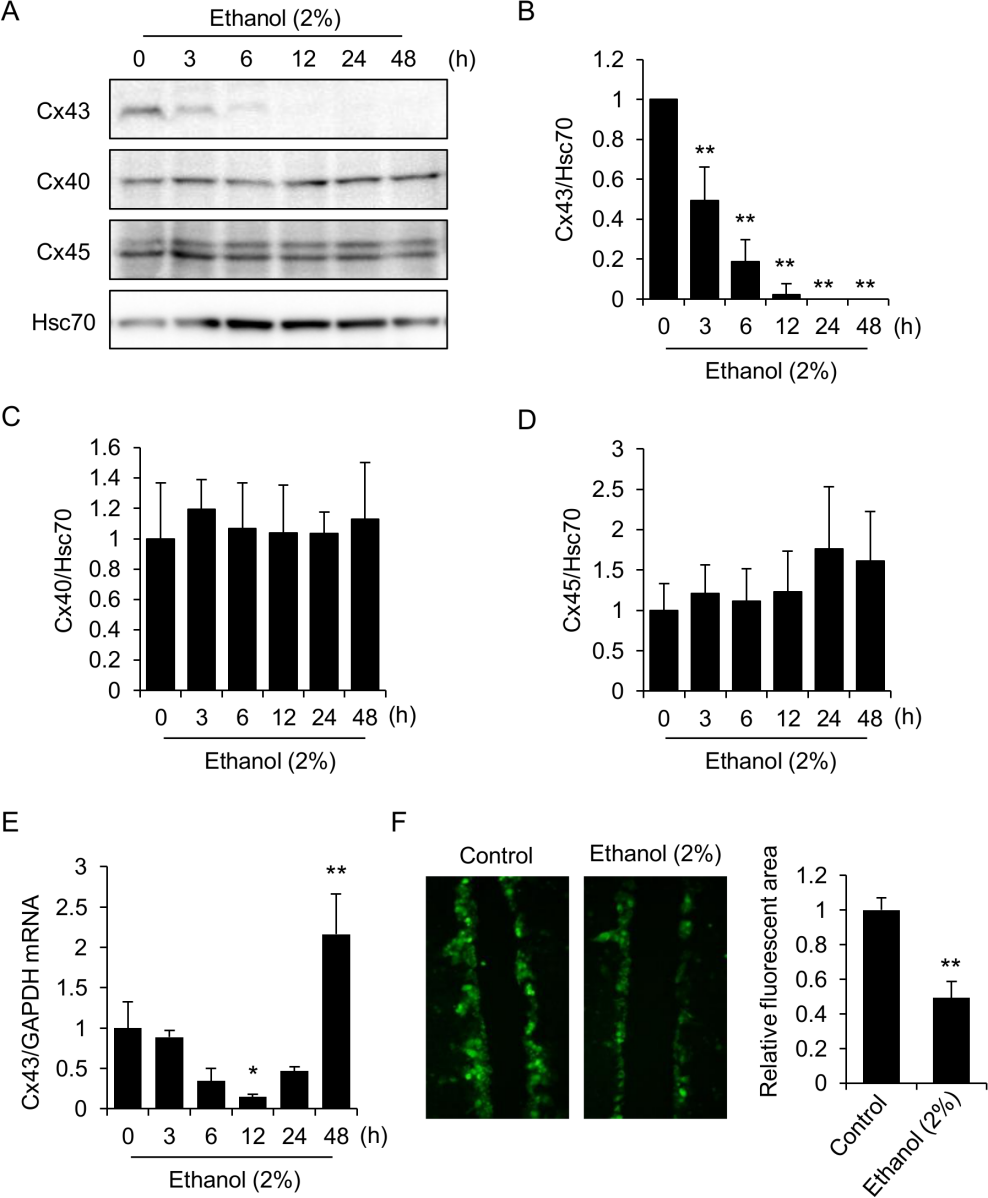
Time-dependent and rapid decrease in Cx43 protein levels in ethanol-treated HL-1 cells. (A-D) Cells were incubated in medium containing 2% ethanol for the indicated time periods, and the protein levels of Cx43, Cx40 and Cx45 were determined by western blot analysis (A). Hsc70 served as a loading control. Quantitative analysis of Cx43 (B), Cx40 (C) and Cx45 (D) is shown. Each graph shows mean±S.E. (n = 4–6). **, p<0.01 versus 0 h. Relative levels to control (0 h) group were shown for Cx43 (B). (E) The levels of Cx43 mRNA were determined by qPCR. GAPDH served as an internal control. The graph shows mean±S.E. (n = 3). **, p<0.01, *, p<0.05 versus 0 h. (F) Ethanol disrupts cell-cell coupling. Cells were incubated in the medium containing 2% ethanol for 48 hours, and scrape-loading dye transfer assay was performed as described in the Materials and methods. Areas of dye spread (fluorescent areas) were quantified using ImageJ software. The graph shows mean±S.E. (n = 5). **, p<0.01 versus control.

Results shown in Figs 1 and 2 prompted us to evaluate changes in the Cx43 protein as well as its transcript, since cell-cell coupling through connexins has been implicated in the propagation of apoptosis [23, 24]. Although the levels of Cx43 in untreated cells varied between the samples (data not shown), probably due to the heterogeneity of HL-1 cells [25], we observed significant and time-dependent decrease of this protein during 2% ethanol exposure (Fig 3A and 3B). The kinetics of Cx43 down-regulation is much faster than that of caspase activation (Fig 2). We next examined the protein levels of other connexins, Cx40 and Cx45, which have been shown expressing in HL-1 cells [25]. In contrast to Cx43, no significant changes were observed in Cx40 and Cx45 levels during ethanol exposure (Fig 3A, 3C, and 3D). qPCR analysis revealed that no significant decrease in Cx43 mRNA was observed after 3 hours of ethanol exposure (Fig 3E). Thus, the rapid down-regulation of the Cx43 protein upon exposure to ethanol (Fig 3A and 3B) should be attributed to its regulation at post-transcriptional levels, implying a facilitated degradation of the Cx43 protein by ethanol exposure. Finally, we performed scrape-loading dye transfer assay to examine whether cell-cell communication was decreased or not upon ethanol exposure. We assessed cell-cell coupling as intercellular spreading of fluorescent dye and found that the dye-spreading areas in ethanol-treated cells decreased significantly compared to control cells (Fig 3F), confirming the impairment of cell-cell coupling in ethanol-treated cells.

### Cx43 is transported into the lysosomes of HL-1 cells exposed to ethanol

**Fig 4:**
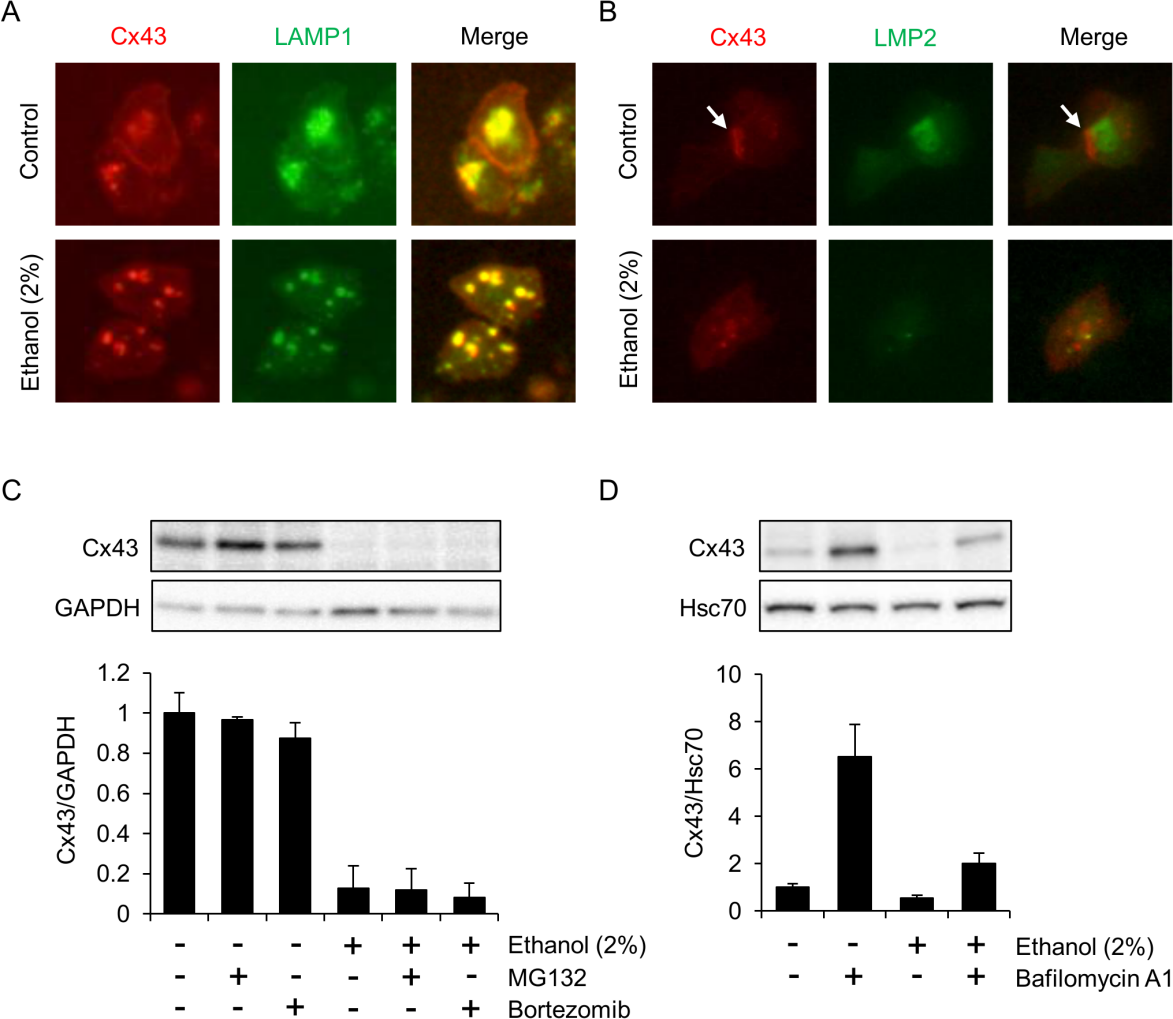
Lysosomal localization of Cx43 in ethanol-treated HL-1 cells. (A and B) Change of Cx43 localization from the plasma membrane to lysosomes in HL-1 cells in response to ethanol exposure. Cells were transfected with combinations of vectors (Cx43-mApple and LAMP1-mGFP for (A) and Cx43-mApple and LMP2-GFP for (B)) and incubated in medium containing 2% ethanol for 24 hours. The cells were then observed under a fluorescence microscope. Arrows indicate gap-junctional cell-cell contact. (C) Proteasome inhibitors do not affect Cx43 protein levels. HL-1 cells were treated with 2% ethanol for 24 hours and Cx43 levels were determined. In some samples, MG132 (500 nM) or bortezomib (5 nM) was included in the medium during the last 6 hours of ethanol exposure. GAPDH served as a loading control. The graph shows mean±S.E. (n = 3). (D) A lysosome inhibitor affects Cx43 protein levels. HL-1 cells were treated with 2% ethanol with or without bafilomycinA1 (50 nM) for 48 hours, and Cx43 levels were determined. The graph shows mean±S.E. (n = 3).

We next examined whether Cx43 is internalized into cytoplasmic compartments, such as lysosomes and proteasomes, for protein degradation upon exposure of HL-1 cells to ethanol. To evaluate the localization of Cx43, we transfected cells with plasmid vectors expressing Cx43-mApple and LAMP1-mGFP. In cells not treated with ethanol (control cells), Cx43-mApple localized on the plasma membrane as well as in the cytoplasm, corresponding to its forward transport from intracellular secretory vesicles into plasma membrane-associated GJs in healthy cardiomyocytes (Fig 4A). Some cytoplasmic Cx43 co-localized with LAMP1 in control cells, implying that lysosomal degradation is occurring for the quick turnover protein even in control cells (Fig 4A). In contrast, almost all of the Cx43 was internalized into the cytoplasm and co-localized with LAMP1 in ethanol-treated cells (Fig 4A). In contrast, the cytoplasmic Cx43 in ethanol-treated cells was found to scarcely co-localize with LMP2, a subunit of the 20S proteasome (Fig 4B). Lysosomal degradation of Cx43 was further confirmed by the distinct effects of proteasome inhibitors (MG132 and bortezomib) and a lysosome inhibitor (bafilomycin A1) on Cx43 levels. Neither MG132 nor bortezomib had any effect on the level of Cx43 protein (Fig 4C), while Cx43 protein levels recovered in the presence of bafilomycinA1 (Fig 4D). Thus, ethanol accelerates the lysosomal degradation of Cx43 in HL-1 cells.

### Evidences against the involvement of autophagy in Cx43 degradation in HL-1 cells during ethanol exposure

**Fig 5:**
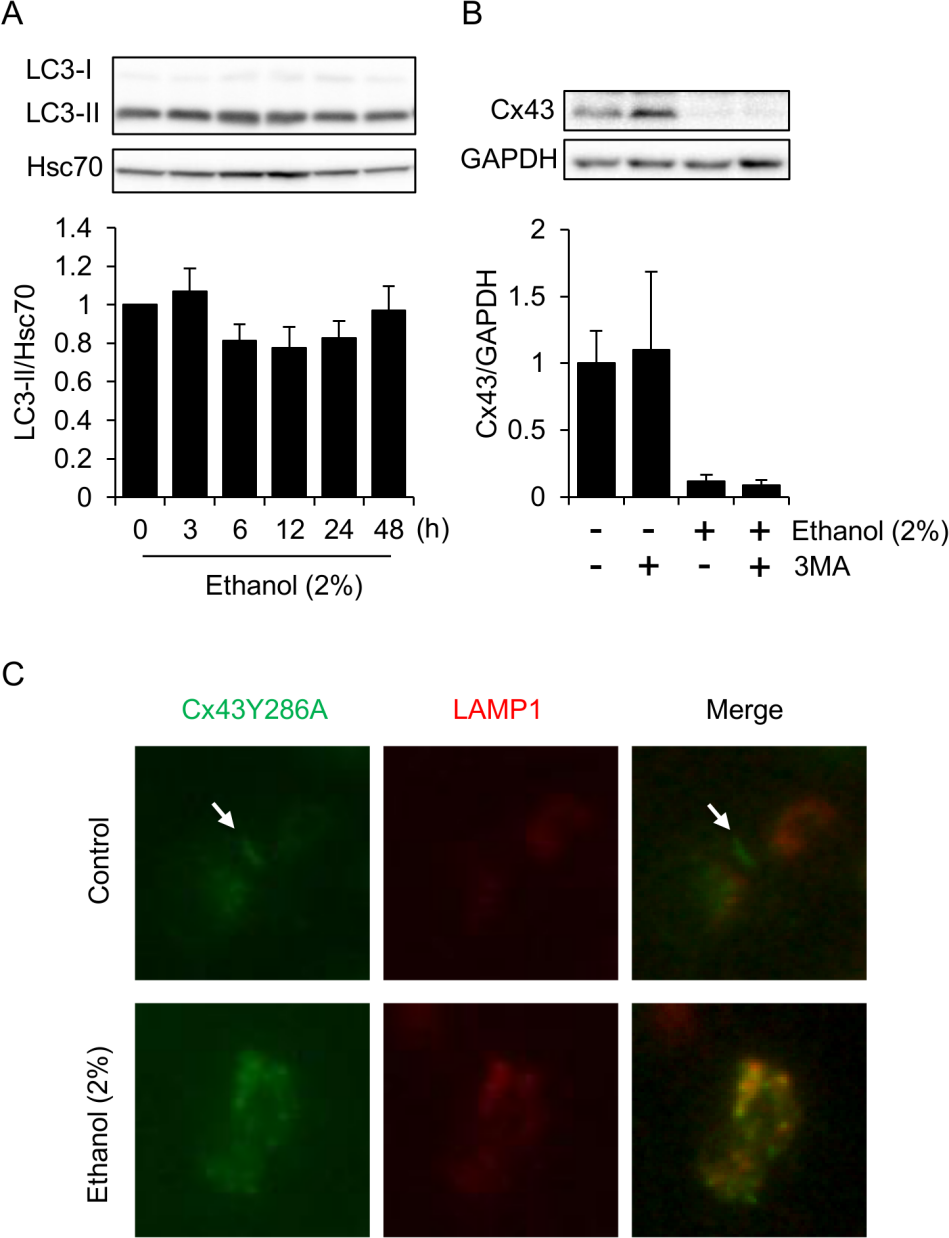
Autophagy is not involved in the degradation of Cx43. (A) LC3-II levels do not increase in HL-1 cells during ethanol exposure. Cells were exposed to 2% ethanol for the indicated time periods, and LC3-II levels were examined by western blot analysis. Hsc70 levels were also examined as a loading control. The graph shows mean±S.E. (n = 3). (B) The inhibition of autophagy by 3MA did not affect Cx43 levels. HL-1 cells were treated with 2% ethanol with or without 5 mM 3MA for 24 hours. Cx43 levels were determined by western blot analysis. The graph shows mean±S.E. (n = 3). (C) Ethanol induces lysosomal localization of a mutant form of Cx43. An expression vector expressing an endocytosis-resistant mutant of Cx43 (Cx43Y286A) fused with GFP was transfected into HL-1 cells together with LAMP1-RFP expression vector, after which the cells were exposed to 2% ethanol for 24 hours and observed under a fluorescence microscope. Arrows indicate gap-junctional cell-cell contact.

The lysosomal degradation of Cx43 prompted us to examine the possible involvement of autophagy in Cx43 degradation. However, we did not detect an increase in the autophagy marker LC3-II at any time during ~48 hours exposure to 2% ethanol (Fig 5A). Furthermore, the phosphoinositide-3 kinase inhibitor 3MA, which is widely used as an autophagy inhibitor [26], had no effect on Cx43 levels in either untreated or ethanol-treated cells (Fig 5B). Furthermore, a Cx43mutant (Cx43Y286A), which is mutated at a tyrosine-dependent sorting signal for clathrin-dependent endocytosis, and is also resistant to autophagic degradation due to its resistance to endocytic internalization [21, 27], was also internalized into cytoplasmic lysosomes upon ethanol exposure (Fig 5C). These results provide evidence against the involvement of autophagy. Moreover, the lysosomal localization of the endocytosis-resistant mutant suggests that lysosomal Cx43 derives not only from the internalization of plasma membrane Cx43, but also from a failure of forward trafficking into the plasma membrane, the latter of which is implicated in the trafficking of Cx43 aberrantly synthesized in stressed cells [28].

### Decrease in the adherens junctional protein N-cadherin as well as the desmosomal protein desomoglein1/2, and the collapse of actin and vimentin structures in HL-1 cells during ethanol exposure

**Fig 6:**
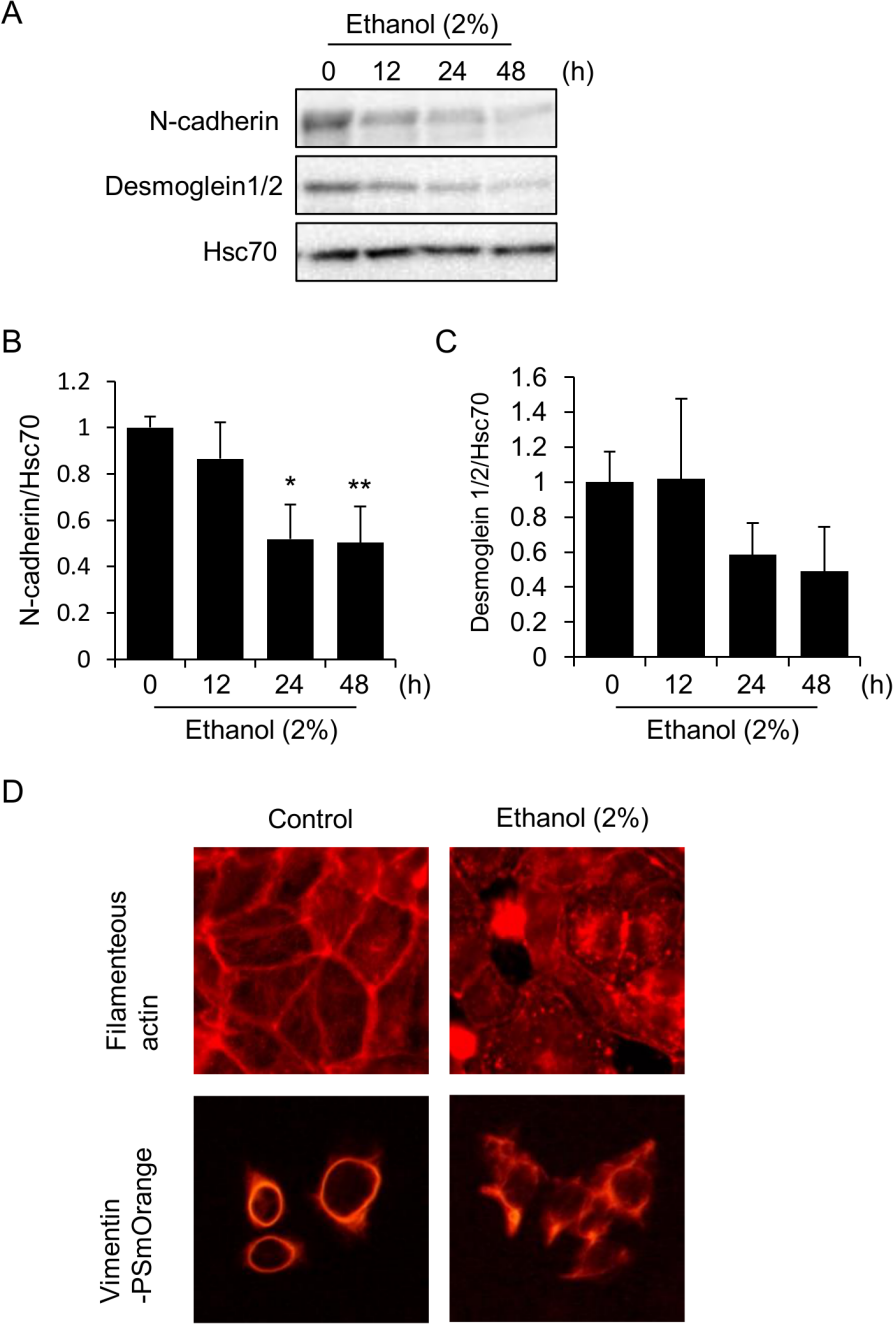
Down-regulation of N-cadherin and desmoglein1/2, as well as rupture of the cytoskeleton in ethanol-exposed HL-1 cells. (A-C) Decreases in the levels of N-cadherin and desmoglein1/2 in HL-1 cells during ethanol exposure. Cells were incubated in medium containing 2% ethanol for the indicated time periods, and the N-cadherin and desmoglein1/2 protein levels were determined by western blot analysis. Hsc70 served as a loading control (A). Quantitative analysis of N-cadherin (B) and desmoglein1/2 (C) is shown. Each graph shows mean±S.E. (n = 3). **, p<0.01, *, p<0.05 versus 0 h. (D) Rupture of the organization of myofibrils and intermediate filaments in ethanol-exposed HL-1 cells. Cells were incubated in medium containing 2% ethanol for 24 hours, stained with phalloidin-rhodamine to visualize filamentous actin, and observed under a confocal fluorescence microscope (upper panel). Cells were also transfected with the vimentin-PSmOrange expression vector, exposed to 2% ethanol for 24 hours, and observed under a fluorescence microscope (lower panel).

We next examined whether ethanol affects the degradation of cell-cell adhesion structural proteins other than Cx43. To examine this, we examined the levels of N-cadherin and desmoglein1/2, the main components of adherens junctions and desmosomes, respectively. Although the kinetics was much slower than that of Cx43 (Fig 3A), N-cadherin showed a time-dependent down-regulation in HL-1 cells upon exposure to ethanol (Fig 6A and 6B). Desomoglein1/2 also showed the tendency to be down-regulated by ethanol exposure (Fig 6A and 6C). Thus, the facilitated degradation of Cx43 is followed by the down-regulation of other ID proteins. We next examined the structures of myofibrils and intermediate filaments. Myofibrils (filamentous actin) were stained with phalloidin-rhodamine while intermediate filaments were visualized by the transfection of the vimentin-PSmOrange expression vector, since cardiomyocytes express vimentin as an intermediate filamentous protein in addition to desmin. The results obtained show that these cytoskeletal structures are normal in control cells: filamentous actin was mainly observed beneath the plasma membrane while intermediate filaments were observed at the periphery of nuclei (Fig 6D). In contrast, diffused staining of these structures was observed in ethanol-exposed cells (Fig 6D). Taken together, these results demonstrate the loss of cell-cell adherence as well as the rupture of cytoskeletal organization in ethanol-treated HL-1 cells.

### Impaired hippo-YAP signaling in ethanol-exposed HL-1 cells

**Fig 7:**
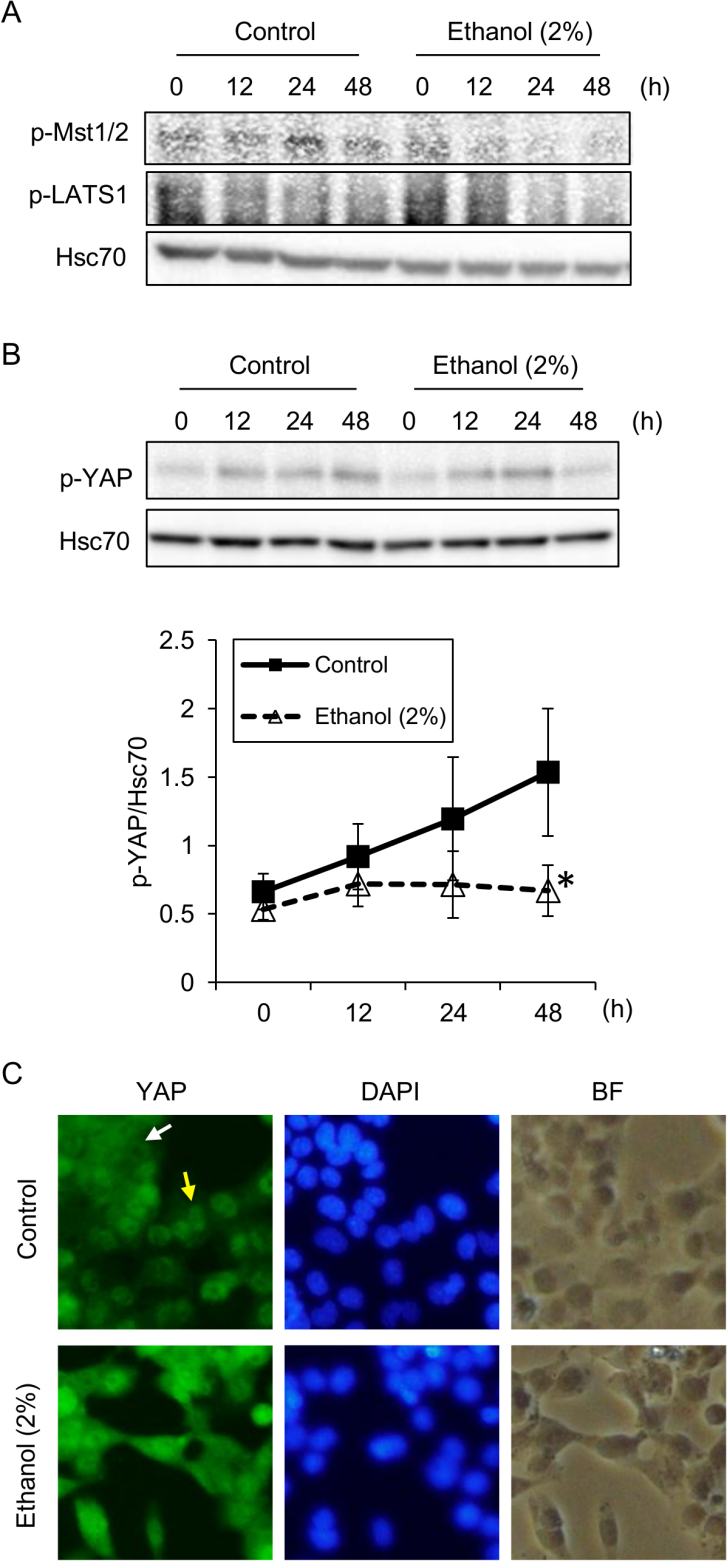
Decreased activation of the hippo pathway and YAP phosphorylation by ethanol in HL-1 cells. (A) Phosphorylation of Mst1/2 and LATS1 and their suppression in HL-1 cells exposed to ethanol. Cells were treated with or without 2% ethanol for the indicated time periods. Levels of phosphorylated Mst1/2 (p-Mst1/2) and phosphorylated LATS1 (p-LATS1) were determined by western blot analysis. Hsc70 served as a loading control. (B) Phosphorylation of YAP and its suppression in HL-1 cells exposed to ethanol. Cells were treated with or without 2% ethanol for the indicated time periods. Levels of phosphorylated YAP (p-YAP) were determined by western blot analysis and normalized to Hsc70 levels. The graph shows mean±S.E. (n = 3). *, p<0.05 versus control. (C) Sub-cellular localization of YAP in cells treated with or without ethanol. Cells were treated with or without 2% ethanol for 24 hours, and the sub-cellular localization of YAP was examined by immunocytochemical analysis under fluorescence microscopy. The white arrow indicates a high-cell-density region where YAP is localized to the nucleus as well as cytoplasm. The yellow arrow indicates a low-cell-density region where YAP is localized to the nucleus.

Since cell-cell contact is prerequisite for hippo signaling, we next evaluated the hippo pathway in HL-1 cells. As shown in Fig 7A, the phosphorylations of both Mst1/2 and LATS1, components of the core hippo pathway machinery, were observed in untreated cells. In contrast, phosphorylations of these two core hippo pathway molecules seemed to be decreased by ethanol exposure (Fig 7A). The hippo pathway effector YAP was phosphorylated in a time-dependent manner in untreated cells, while its phosphorylation was significantly decreased compared to control cells after 48 hours of ethanol exposure (Fig 7B). Hence, ethanol seems to suppress hippo signaling in HL-1 cells. It has been reported that the decreased phosphorylation of YAP leads to the nuclear translocation of this transcriptional co-activator protein; therefore, we examined the sub-cellular localization of YAP in cells exposed to ethanol. Without ethanol exposure, cell cultures consisted mainly of high-cell-density regions where YAP was localized to the cytoplasm as well as the nucleus, and the nuclear localization of YAP was observable in low-cell-density regions (Fig 7C). Although the cell cultures consisted mainly of low-cell-density regions after ethanol exposure, due to loss of the cells by apoptosis, there was no obvious nuclear localization of YAP observed as compared with control cells (Fig 7C). These results suggest that decreased phosphorylation of YAP was not followed by increased nuclear translocation. Thus, the hippo-YAP signaling axis might be disrupted to some extent in ethanol-exposed cells.

### Implications of cell-cell communication and the hippo pathway in HL-1 cell damage by ethanol

**Fig 8:**
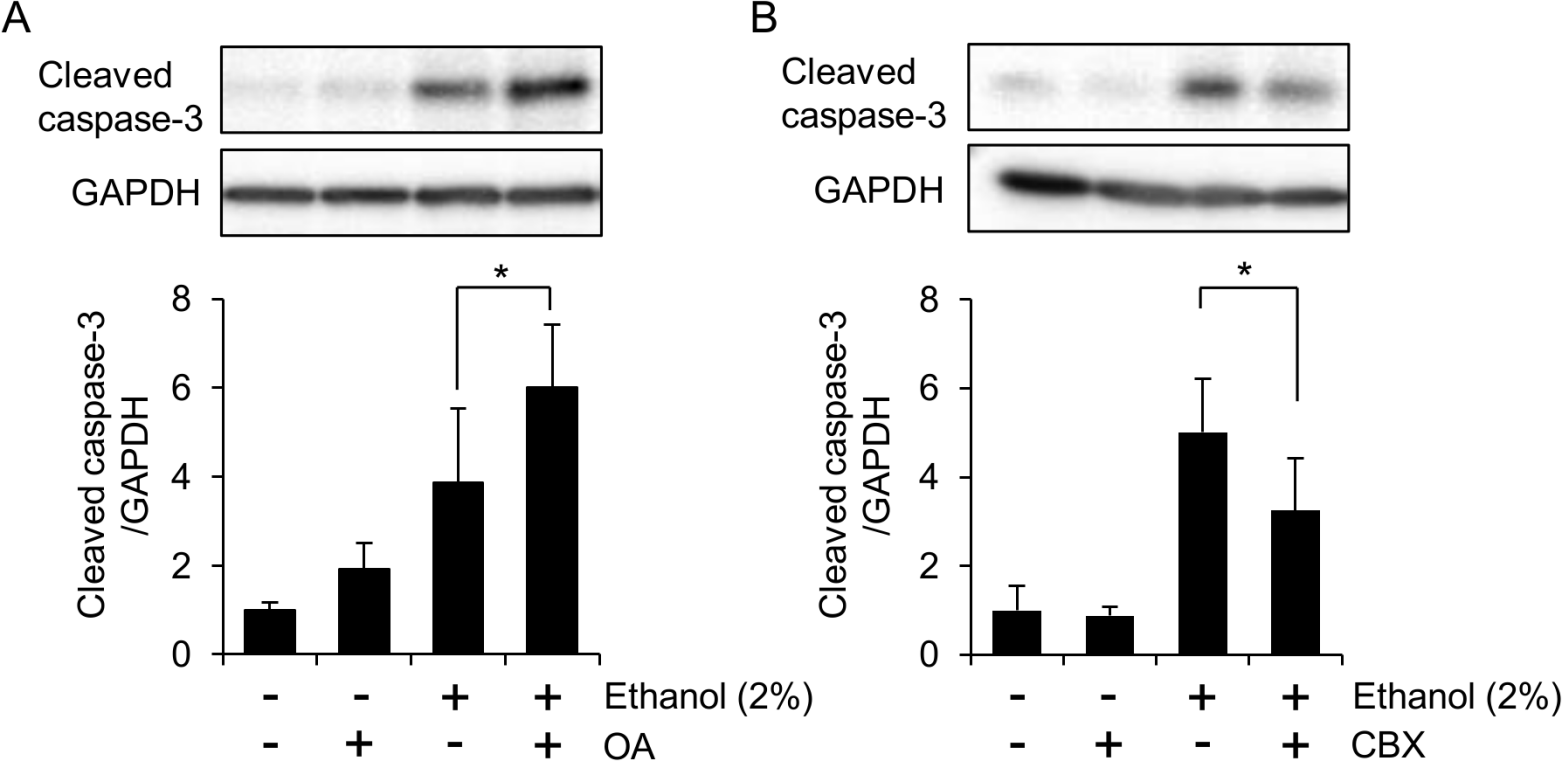
Effects of okadaic acid and carbenoxolone on ethanol-induced apoptosis in HL-1 cells. (A) Okadaic acid (OA) mitigates ethanol-induced apoptosis in HL-1 cells. Cells were treated with OA (10 nM), ethanol (2%), or both for 48 hours, and caspase-3 activation was assessed by western blot analysis. The graph shows mean±S.E. (n = 6). *, p<0.05. (B) Carbenoxolone (CBX) suppresses ethanol-induced apoptosis in HL-1 cells. Cells were treated with CBX (100 μM), ethanol (2%), or both for 48 hours, and caspase-3 activation was assessed by western blot analysis. The graph shows mean±S.E. (n = 5). *, p<0.05.

Finally we evaluated the significance of hippo pathway in cardiac damage using okadaic acid (OA), a protein phosphatase inhibitor that selectively inhibits PP2A at low concentrations [29]. The attenuation of PP2A activity by OA has been shown to result in the activation of the hippo pathway, making this substance suitable for use as a hippo pathway activator [29]. As shown in Fig 8A, treatment with OA (10 nM) alone caused slight activation of caspase-3 in HL-1 cells, suggesting that activation of the hippo pathway is detrimental to ethanol-treated cells. The effects of OA and ethanol on caspase-3 activation seemed to be additive (Fig 8A). Thus, the tendency of hippo pathway supression in ethanol-exposed cells might contribute to the maintenance of cellular homeostasis. In contrast, the administration of a GJ inhibitor, carbenoxolone (CBX), significantly decreased the caspase-3 activation by ethanol exposure (Fig 8B), indicating that cell-cell communication through GJ might be detrimental for cellular homeostasis during direct exposure to ethanol. Taken together, both the facilitated degradation of Cx43 and the decreased tendency of hippo pathway activation appear to be processes by which cells maintain homeostasis, rather than propagating damage.

## Discussion

The down-regulation and aberrant localization of the GJ protein Cx43 has been repeatedly implicated in the pathophysiology of heart failure [30], as results from ischemic heart disease [31] and intoxication by cardiotoxic drugs [32]. Decreased ratio of Cx43/Cx40 protein levels has also been reported in an experimental animal model of congestive heart failure [33] and the decrease of Cx43/Cx40 ratio has been suggested to reduce intracellular transfer of Lucifer Yellow *in vitro* [34]. Our current results are matched well with these reports but the molecular mechanism underling selective degradation of Cx43 remains to be elucidated. Lai et al. reported that in the mice given ethanol-containing water for twelve weeks, Cx43 levels increased, but that its distribution was disorderly [35]. Although our current data show a severe reduction in Cx43 protein levels during acute and direct exposure of HL-1 cells to ethanol (Fig 3A), Cx43 gene expression tended to recover after 24–48 hours of ethanol exposure (Fig 3E). This biphasic response of Cx43 has also been reported, for example, in human collecting duct cells treated with TGF-β[36]. Since Cx43 gene expression is regulated through multiple mechanisms by transcription factors, microRNAs, and epigenetic factors [37], mechanism responsible for this biphasic Cx43 gene expression should be investigated in future studies.

In any case, the recovery of Cx43 gene expression suggests that cellular reactions toward remodeling/regeneration of the GJ might occur, though we could not observe any recovery of Cx43 protein under our experimental conditions. There are conflicting results concerning the role of Cx43 in apoptosis. For example, Cx43 has been reported to reduce ischemic neuronal cell death [38], while it propagates spontaneous apoptosis in hepatocytes [23, 39]. Interestingly, it has been reported that Cx43 moves to the mitochondria during ischemic preconditioning in mammalian heart [40]. However, we observed no mitochondrial localization of Cx43 in ethanol-exposed HL-1 cells (data not shown). The amelioration of ethanol-induced apoptosis by the GJ inhibitor CBX (Fig 8B) suggests a detrimental role of GJ in ethanol-exposed HL-1 cells. We observed a tendency that the induction of apoptosis by ethanol might be affected by cell confluency (data not shown). This observation is coincided to the fact that the spontaneous contractile phenotype of HL-1 cells is crucially governed by the level of confluency [13].

Once an organ or tissue is fully developed, the hippo pathway is activated and phosphorylates YAP, which is then degraded in the cytoplasm [10]. However, YAP could be reactivated to regenerate injured tissues/organs when hippo signaling decreases due to a loss of cell-cell contact [11, 41]. Indeed, the forced expression of YAP has been shown to regenerate fully differentiated adult cardiomyocytes following damage due to myocardial infarction [42]. It has also been reported that hippo pathway-deficient adult cardiomyocytes can enter a proliferation cycle for regeneration [41]. Our results show that ethanol-induced disruption of cell-cell contact (Figs 3A and 6A) leads to decreased phosphorylation of YAP (Fig 7B). The hippo pathway activator OA had an additive effect on ethanol-induced apoptosis (Fig 8A), further confirming that down-regulation of the hippo pathway helps to protect the heart against ethanol-induced cardiotoxicity. However, decreased YAP phosphorylation does not seem to cause in efficient nuclear translocation of YAP (Fig 7C). This might be due to the loss of mechanotransduction through actomyosin tension in ethanol-exposed cells. Actomyosin tension is another activator of YAP in addition to the hippo pathway [43], but should be impaired due to the disruption of cytoskeletal organization in ethanol-exposed cells (Fig 6D). Thus, our current results demonstrate possible roles of the impairment of hippo-YAP signaling in ethanol cardiotoxicity.

Ma, et al. have suggested that PP2A levels are significantly elevated in the hearts of mice administered ethanol intraperitoneally [44]. The down-regulation of the hippo pathway in ethanol-exposed cells (Fig 7) is consistent with this report, since PP2A has been shown to be a potent suppressor of the hippo pathway [45]. Interestingly, PP2A has also been shown to be associated with Cx43 in failing hearts [46]. Thus, PP2A might be involved in the facilitated degradation of Cx43 as well as in the down-regulation of hippo signaling in ethanol-exposed cells. In addition to the possible role of PP2A in ethanol-exposed cardiomyocytes, a recent study has shown that LATS1 is a target of the E3 ubiquitin ligase NEDD4 [47]. NEDD4 is also required for Cx43 ubiquitination and subsequent degradation [8]. Thus, there is a possibility that NEDD4 is also involved in the degradation of Cx43 and suppression of the hippo pathway.

In conclusion, this study is, to the best of our knowledge, the first report showing the involvement of the hippo-YAP signaling axis in the cellular degeneration caused by ethanol, other than a report that indicated a relationship between the hippo pathway and tissue growth during chronic ethanol feeding in *Drosophila* [48]. Our results shed light on the potential importance of the hippo-YAP signaling axis in ethanol-induced cardiotoxicity.
